# Dynamic alterations in metabolomics and transcriptomics associated with intestinal fibrosis in a 2,4,6-trinitrobenzene sulfonic acid-induced murine model

**DOI:** 10.1186/s12967-023-04392-0

**Published:** 2023-08-18

**Authors:** Jinzhen Wu, Zhenyi Tian, Xiaoduan Zhuang, Yiru Chen, Tingting Fan, Jiayun Li, Xinying Wang

**Affiliations:** Department of Gastroenterology, Zhujiang Hospital, Southern Medical University, No.253, Industrial Avenue, Haizhu District, Guangzhou, 510000 Guangdong People’s Republic of China

**Keywords:** Inflammatory bowel disease, Intestinal fibrosis, RNA-sequencing, Metabolomics, Lipid metabolism

## Abstract

**Background & aims:**

Intestinal fibrosis is a common and severe complication of inflammatory bowel disease without clear pathogenesis. Abnormal expression of host genes and metabolic perturbations might associate with the onset of intestinal fibrosis. In this study, we aimed to investigate the relationship between the development of intestinal fibrosis and the dynamic alterations in both fecal metabolites and host gene expression.

**Methods:**

We induced intestinal fibrosis in a murine model using 2,4,6-trinitrobenzene sulfonic acid (TNBS). TNBS-treated or control mice were sacrificed after 4 and 6 weeks of intervention; alterations in colonic genes and fecal metabolites were determined by transcriptomics and metabolomics, respectively. Differential, tendency, enrichment, and correlation analyses were performed to assess the relationship between host genes and fecal metabolites.

**Results:**

RNA-sequencing analysis revealed that 679 differential genes with enduring changes were mainly enriched in immune response-related signaling pathways and metabolism-related biological processes. Among them, 15 lipid metabolism-related genes were closely related to the development of intestinal fibrosis. Moreover, the fecal metabolic profile was significantly altered during intestinal fibrosis development, especially the lipid metabolites. Particularly, dynamic perturbations in lipids were strongly associated with alterations in lipid metabolism-related genes expression. Additionally, six dynamically altered metabolites might serve as biomarkers to identify colitis-related intestinal fibrosis in the murine model.

**Conclusions:**

Intestinal fibrosis in colitis mice might be related to dynamic changes in gene expression and metabolites. These findings could provide new insights into the pathogenesis of intestinal fibrosis.

**Supplementary Information:**

The online version contains supplementary material available at 10.1186/s12967-023-04392-0.

## Background

Inflammatory bowel disease (IBD), including Crohn’s disease (CD) and ulcerative colitis (UC), has become a global public health challenge owing to its increasing incidence [1]. Intestinal fibrosis is a frequent long-term complication of IBD that often results in functional damage and bowel stenosis requiring surgical intervention, particularly in patients with CD [2]. To date, a poor understanding of its pathogenesis has hampered the clinical management of patients and development of effective anti-fibrotic therapies [2]. Moreover, the presence and degree of fibrosis or stenosis cannot be predicted by cross-sectional imaging, endoscopy, or histology [3]. Thus, there remains a pressing need to elucidate the dynamic pathogenesis of intestinal fibrosis. However, a major obstacle for understanding the pathogenetic mechanisms of intestinal fibrosis in IBD patients is that its early and ensuing time-dependent phases cannot be tracked [2]. Therefore, well-characterized animal models of intestinal fibrosis have been widely used to reproduce the fibrogenic pathological process to reveal pathogenesis [4].

Intestinal fibrosis is a heterogeneous process involving multiple intricate and interacting mechanisms that include aberrant immune and non-immune responses, host-microbiome interactions, mesenteric adipocytes, and genetic susceptibility [5, 6]. Studies have indicated that genetic variants encoding immunomodulatory proteins, pro- and anti-inflammatory cytokines, and fibrogenic factors are associated with the fibrostenotic phenotype of CD [3]. Some studies have suggested that the abnormal expression of certain genes, such as nucleotide-binding oligomerization domain 2 (*NOD2*), toll-like receptors-4 (*TLR4*), and tumor necrosis factor-like ligand 1A (*TL1A*), might be related to the development of intestinal fibrosis [5, 7]. These genes play a role in intestinal fibrogenesis via regulating certain important processes, including epithelial-mesenchymal transition (*EMT*) initiation and progression, fibrogenic signal transduction, and macrophage-fibroblast program [7–9]. However, current studies have failed to identify the relationship between the dynamic fluctuation of gene expression and colitis-related intestinal fibrosis. Hence, we sought to profile the dynamic gene expression during intestinal fibrosis by time-course RNA-sequencing (RNA-seq) in a murine model, which could provide important information for studying the underlying mechanisms of colitis-related intestinal fibrosis.

The close and complex connections between genes and metabolites have contributed to the development of multiple diseases. Subtle changes in protein-coding genes (particularly those encoding metabolic enzymes) can lead to 10,000-fold changes in metabolite abundance [10]. Mechanistic studies have revealed that metabolism-related genes can promote fibrosis in other organs by regulating metabolic processes that are strongly linked with fibrosis development, such as fatty acid metabolism [11]. Metabolites can also serve as active modulators of gene activity by controlling transcription factors and performing post-transcriptional modifications, to modulate biological processes and phenotypes [12]. Moreover, metabolites participate in the pathogenesis of multiple diseases by acting as signaling molecules, immune modulators, endogenous toxins, and environmental sensors [10]. In particular, changes in tricarboxylic acid cycle intermediates, amino acid and lipid metabolism products, as well as oxidative metabolites are closely associated with energy metabolism, intestinal barrier, immune system, and disease activity in patients with IBD [13–15]. Additionally, emerging evidence suggests that alterations in metabolism are not only a feature of fibrosis but may also play an influential role in its pathogenesis [16–18]. Recently, in vivo studies have shown that leptin and trimethylamine N-oxide can promote the fibrosis process in various organs, including the liver, lung, heart, and kidney [19–21]. Macias et al. [22] reported increased serum succinate levels and colonic succinate receptor (*SUCNR1*) expression in CD patients and demonstrated the role of *SUCNR1* in murine colitis and intestinal fibrosis. However, the dynamically altered metabolites and their effects on intestinal fibrosis remain obscure. Thus, a comprehensive genetic and metabolic analysis might elucidate the potential roles of altered genes and metabolites in the development of intestinal fibrosis.

In this study, the 2,4,6-trinitrobenzene sulfonic acid (TNBS) model, a classic murine model [23], was employed to reproduce and evaluate the fibrogenic pathological process at different stages of chronic colitis-related intestinal fibrosis model. RNA-seq was conducted to identify the underlying genetic changes in colonic samples. Simultaneously, fecal widely-targeted metabolomics were performed to investigate the dynamic metabolite disturbances and screen for associated metabolic markers. Integrated analysis combining RNA-seq and targeted metabolomics was then performed to identify correlations between host genes and metabolites that were associated with morbid conditions. Our findings provide new insights into the pathogenesis of intestinal fibrosis.

## Methods

### Induction of intestinal fibrosis

The animal experimental protocol was approved by the Institutional Animal Care and Use Committee of the Zhujiang Hospital of Southern Medical University (Guangzhou, China). We randomly divided 15 male 8-week-old C57BL/6 mice into three groups (Control, TNBS-4W, and TNBS-6W groups, *n* = 5 per group). All mice received weekly intra-rectal administration of TNBS solution (Sigma-Aldrich, USA) or vehicle for 6 weeks as previously described [24, 25]. Briefly, after fasting for 12 h, mice were anesthetized and treated with 0.1 mL of an increasing dose of TNBS solution (in 45% ethanol) or 45% ethanol. The TNBS enema concentrations from the first to sixth week were 0.75% (*w/v*), 1.0% (*w/v*), 1.5% (*w/v*), 2.0% (*w/v*), 2.0% (*w/v*), and 2.5% (*w/v*), respectively. The feces of mice were collected on day 2 after the fourth and sixth enemas and frozen in liquid nitrogen for metabolites detection. Mice were anesthetized and then sacrificed on day 3 after the fourth and sixth doses, and the colons were harvested for RNA extraction and histological staining.

### Histological assessment and quantitative polymerase chain reaction (qPCR)

After sample collection, colons were frozen in liquid nitrogen for RNA extraction, or fixed in 4% paraformaldehyde. The fixed colons were then dehydrated in gradient ethanol, embedded in paraffin, sliced into 4-μm-thick sections, and subjected to Masson’s trichrome and hematoxylin and eosin (H&E) staining. Subsequently, collagen deposition (blue staining) was quantified using the ImageJ software (National Institutes of Health).

The expression of fibrotic indicators in colons was determined by qPCR. Briefly, total RNA was extracted from colons using the TRIzol reagent (Takara, Japanese) and then converted to cDNA using the reverse transcriptase kit (Accurate Biology, China). Subsequent qPCR was performed on the CFX Connect real-time PCR detection system (Bio-Rad Laboratories, Hercules, CA, USA) with a SYBR Green Pro Taq HS Premix (Accurate Biology, China). The primer sequences are shown in the Additional file 1: Table S1.

### RNA-seq and data processing

RNA integrity and purity were assessed prior to cDNA library preparation. Samples with concentrations > 50 ng/μL, RIN > 7.0, and total RNA > 1 μg were used in downstream experiments. Briefly, mRNA was isolated according to the polyA selection method using oligo (dT) beads (Cat. 25–61005, Thermo Fisher Scientific, USA) and then fragmented using a magnesium ion interruption kit (Cat. E6150S, USA). Double-stranded cDNA was synthesized using reverse transcriptase (Cat. 1896649, CA, USA), *E. coli* DNA polymerase I (Cat. m0209, USA), and RNase H (Cat. m0297, USA). After end repair, 3′ adenylation, adaptation ligation, and UDG enzyme treatment, the double strands were pre-denatured by PCR to form a 300 ± 50 bp chain library. The paired-end RNA-seq library was sequenced using an Illumina Novaseq 6000 PE150 platform (LC Bio Technology, Hangzhou, China).

Raw sequencing data were processed using the FastQC software, and clean reads were aligned to mouse reference genomes (GRCm38) using the HISAT2 software with default parameters. The mRNA levels were quantified as the value of fragments per kilobase of exon per million mapped reads (FPKM). The edgeR package [26] was used to identify differentially expressed genes (DEGs) among the three experimental groups according to the screening criteria *q* < 0.05. The Short Time-series Expression Miner (STEM) [27] was used to classify the identified DEGs expression patterns. The online platform AnimalTFDB v4.0 [28] was used for transcription factors (TFs) and transcription cofactors (TcoFs) analysis. Gene Ontology (GO) and Kyoto Encyclopedia of Genes and Genomes (KEGG) pathway enrichment analyses were conducted using the pathview [29] and clusterProfiler [30] packages to determine the functions of the DEGs(species: Mus musculus). The enrichment analysis results with P < 0.05 were further analyzed. Correlations between the screened genes and fibrotic indicators were then assessed using Spearman’s correlation analysis.

### Targeted metabolomics profiling and data processing

After thawing on ice, 20 mg of fecal sample was mixed with 400 μL of 70% methanol–water internal standard extractant, vortexed for 3 min, and sonicated for 10 min in an ice water bath. Homogenization and sonication cycles were repeated twice, followed by incubation at − 20 °C for 30 min and centrifugation at 12,000 × *g* for 10 min at 4 °C. The supernatants were transferred to LC–MS vials and stored at − 80 °C until UPLC–MS/MS analysis. The sample extracts were analyzed using an LC-ESI–MS/MS system xionLC AD (UPLC, E, https://sciex.com.cn/; MS, QTRAP® System, https://sciex.com/). Based on the self-built target database of Matteville Biotechnology Co., Ltd. (Wuhan, China), qualitative analysis was performed according to the retention time, parent ion pair information, and secondary spectrum data. Quantification was performed using the multiple reaction monitoring mode of triple quadrupole mass spectrometry. The identified metabolites were annotated using the KEGG compound database. Mass spectrometry data were processed using the Analyst 1.6.3 software.

The raw data was log transform (log2) and mean centering before partial least squares discriminant analysis (PLS-DA). A permutation test (200 permutations) was performed to avoid overfitting. The significance of metabolites was determined using the variable importance projection (VIP) value of the PLS-DA model combined with the fold-change (FC) value of univariate analysis. Metabolites with VIP ≥ 1 and absolute log2FC (|log2FC|) > 0.58 were considered to be significantly altered metabolites (SAMs). Venn diagram and classification pie chart were used to identify the common SAMs. The SAMs content was standardized, and k-means clustering analysis and cluster heatmap were then performed to study the metabolite trends between samples. A random forest algorithm in machine learning was completed to select the characteristic material, and its performance was subsequently evaluated using the receiver operating characteristic (ROC) curve analysis module of the online platform MetaboAnalyst5.0 (https://www.metaboanalyst.ca). Correlations between the screened metabolites and fibrotic indicators were determined using Spearman’s correlation analysis.

### Integrated analysis of transcriptomics and metabolomics

Integrated metabolic pathway analysis of transcriptomics and metabolomics data was performed on the MetaboAnalyst5.0 platform. After identifying the DEGs enriched in lipid metabolism-related biological processes, the STRING database and Cytoscape plug-in CytoHubba were used to perform protein–protein interaction (PPI) network prediction and screen the top15 hub genes. Correlations between the screened genes and metabolites were determined using Spearman’s correlation analysis.

### Statistical analysis

Statistical analyses were conducted using the GraphPad Prism 9.0.1 (GraphPad Software, CA, USA). The results were assessed using one-way analysis of variance (ANOVA) and expressed as the mean ± standard error of mean (SEM). In datasets operated by ANOVA, the Bonferroni post-hoc test was adopted. The parameters of all the clustering correlation heatmap with signs were set as followed: cluster methods: complete, cluster distance: Euclidean, correlation methods: Spearman. Statistical significance was set at *P* < *0.05*.

## Results

### Continuing TNBS administration promotes the development of intestinal fibrosis in IBD model

**Fig. 1:**
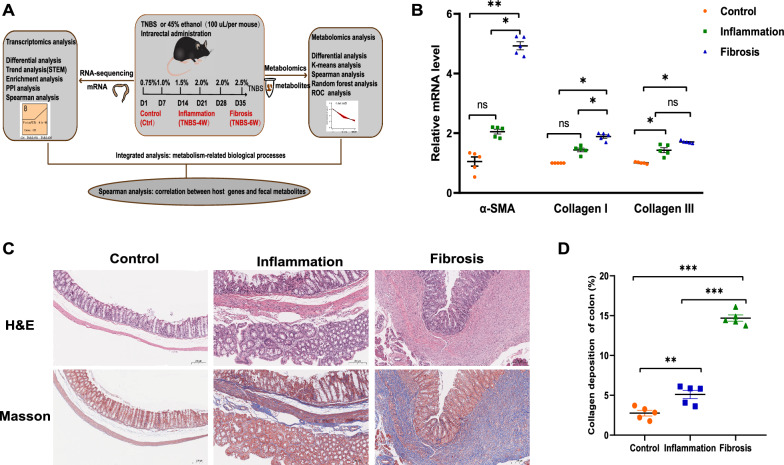
Continuing TNBS rectal administration promotes the development of intestinal fibrosis. **A** Experimental workflow used to characterize the dynamic changes in gene and metabolite profiles during the progression of intestinal fibrosis. **B** Relative mRNA level of fibrotic indicators (α-SMA, collagen I, and collagen III). Data are presented as the mean ± SEM  (*n* = 5 per group). **C** Representative images of Masson’s trichrome and H&E staining of colon sections from C57BL/6 mice (scale bar = 200 µm). **D** Quantification of collagen deposition (trichrome blue staining) was measured in three representative areas per sample using ImageJ software (*n* = 5 per group). N = 3, *ns* not significant, *: P < 0.05, **: P < 0.01, ***: P < 0.001. *H&E* hematoxylin and eosin

We used a TNBS-induced mouse model of intestinal fibrosis to characterize gene and metabolite changes during the progression of chronic inflammation-related intestinal fibrosis (Fig. 1A). Colons and feces were collected from mice after the fourth (TNBS-4W or Inflammation) and sixth (TNBS-6W or Fibrosis) enema, according to the stage of intestinal fibrosis in the IBD model [24, 25, 31]. A marked increase in fibrotic indicators, including α-SMA, collagen I, and collagen III, was observed over the course of TNBS treatment (Fig. 1B). Masson’s trichrome staining showed increased deposition of collagen in the mucous layer and submucosa and thickening of the intestinal wall (Fig. 1C, D), while H&E staining revealed obvious inflammatory infiltration at the collagen deposition site (Fig. 1C). The above results were consistent with previous reports [24, 31–33], indicating that serial TNBS administration can successfully trigger fibrotic reactions in mice. In addition, these pathological results were similar to the histological manifestations of fibrosis-related stenosis in patients with IBD [34], suggesting that our model is suitable and reliable for studies of colitis-related intestinal fibrosis. Therefore, this model could provide valuable information for revealing the pathogenesis of intestinal fibrosis.

### Aberrant gene expression profiles in the development of intestinal fibrosis

**Fig. 2:**
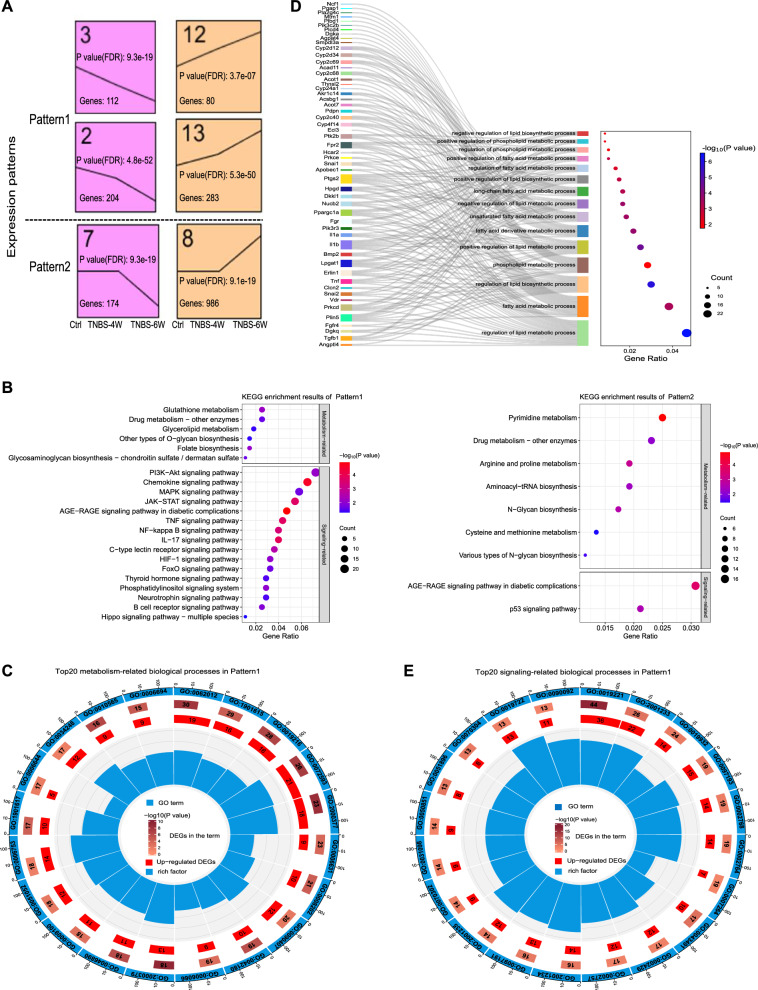
Time-course RNA-seq analysis of colonic tissues from the fibrosis mouse model. **A** Significant gene expression patterns identified by the STEM software; all expression patterns follow the timeline of Control (Ctrl), Inflammation (TNBS-4W), and Fibrosis (TNBS-6W). **B** KEGG enrichment results (mainly including signaling and metabolism-related pathways) of Pattern1 and Pattern2 (*P* < 0.05), in which Gene Ratio indicates the proportion of DEGs enriched in each pathway to the total number of DEGs, the size of the circle represents the number of genes enriched in the pathway, and the color of the circle represents the significance of enrichment. **C** The top20 metabolism-related biological processes in Pattern1 (*P* < 0.05, ranked by the number of DEGs in each enriched term). **D** GO enrichment results for terms related to lipid metabolic processes, wherein the size of the circles represents the number of DEGs in each enriched term and color of the circles represents the significance of enrichment (*P* < 0.05). **E** The top20 signaling-related biological processes in Pattern1 (*P* < 0.05, ranked by the number of DEGs in each enriched term). GO enrichment results are presented as circle diagrams: the first circle (from outside to inside) represents the GO term, and outer circle represents the coordinate scale of the number of genes; second circle represents the number of DEGs annotated to the GO entry, and color represents the -log10 (*P* value) of the enrichment analysis; third circle represents the number of differentially up-regulated DEGs in the term; and fourth circle represents the percentage of enrichment factors. *n* = 5 per group. *STEM* Short Time-series Expression Miner, *KEGG* Kyoto Encyclopedia of Genes and Genomes; *GO* Gene Ontology, *DEGs* differentially expressed genes

To investigate the mechanisms underlying intestinal fibrosis development, we conducted a time-course RNA-seq analysis using colons of the 15 mice. A total of 2370 DEGs (*q* < 0.05), all of which were expressed to different degrees in the three groups, were screened (Additional file 1: Table S2). Six significant genetic profiles were revealed by the false discovery rate method with at least two-fold expression changes set between the maximum and minimum and 1000 permutations (Additional file 1: Table S3). The six profiles were categorized into two patterns according to the time at which they were considerably up- or down-regulated (Fig. 2A). Pattern1 contains continuously responsive genes that consistently increased or decreased during fibrogenesis progression. Pattern2 reflects late-stage responsive genes that were significantly enhanced or reduced after four doses of TNBS. The 1839 DEGs in Pattern1 and Pattern2 were described in the Additional file 1: Table S4. Since TFs and TcoFs play crucial roles in regulating gene expression and all kinds of biological processes, 82 TFs and 109 TcoFs were further identified in Pattern1 and Pattern2 (Additional file 1: Table S5-S6). Among them, 13 TFs were consistently decreased (*e.g.*, vitamin D receptor (*Vdr*) and forkhead box O3 (*Foxo3*)) while 19 TFs were consistently increased (*e.g.*, snail family zinc finger 1 (*Snai1*), *Snai2* and myelocytomatosis oncogene (Myc)).

To elucidate which pathways are predominant in intestinal fibrosis formation, we next conducted functional enrichment analysis for the DEGs. KEGG enrichment analysis showed that the genes in Pattern1 were enriched in metabolism-related pathways, such as glutathione metabolism, folate biosynthesis, glycerolipids metabolism, and other types of O-glycan biosynthesis (Fig. 2B). Genes in Pattern2 were mainly enriched in pyrimidine metabolism, arginine and proline metabolism, aminoacyl-tRNA biosynthesis, cysteine and methionine metabolism, and N-glycan biosynthesis (Fig. 2C). GO enrichment analysis revealed that 52 DEGs in Pattern1 were enriched in multiple biological processes related to lipid metabolism, including fatty acid, steroid, and phospholipid metabolic processes (Fig. 2C, D, Additional file 1: Table S7). Interesting, among them, *Vdr*, *Snai2* and *Snai1* are remarkable TFs, while transforming growth factor, beta 1 (*Tgb1*), peroxisome proliferative activated receptor gamma coactivator 1-alpha (*Ppargc1a*) and diacylglycerol kinase, theta (*Dgkq*) are significant TcoFs. The genes in Pattern1 were also enriched in the metabolic processes of other substances, including nucleoside phosphate metabolic, organic hydroxyl compound biosynthetic, reactive oxygen species metabolic, cellular ketone metabolic, and alcohol metabolic (Fig. 2C). Interestingly, most genes in these metabolism-related biological processes were up-regulated after TNBS treatment, indicating strong metabolic activation in the progression of intestinal fibrosis (Fig. 2C, Additional file 1: Table S8). Therefore, we hypothesized that metabolic reprogramming is associated with intestinal fibrosis progression.

Genes involved in signal transduction play an important role in the development of fibrosis [31–33]. KEGG enrichment analysis suggested that the genes in Pattern1 were enriched in pathways associated with immune responses, such as phosphatidylinositol 3-kinase/protein kinase B (PI3K-Akt), mitogen-activated protein kinase (MAPK), Janus kinase/signal transducers and activators of transcription (JAK-STAT), tumor necrosis factor (TNF), interleukin-17 (IL-17), and NF-kappa B signaling pathways (Fig. 2B). Meanwhile, the dominant biological processes in Pattern1 were associated with cytokine-mediated, second-messenger-mediated, apoptotic, and immune response-regulating signaling pathways (Fig. 2E, Additional file 1: Table S8). Based on these results, we hypothesized that colitis-related intestinal fibrosis is primarily mediated through modulation of immune response and metabolism-related pathways.

### Alterations of fecal metabolites in the development of intestinal fibrosis

Metabolites are the downstream products of multiple intracellular actors, including genes, transcriptional activators, and RNA transcripts [10]. Thus, fecal targeted metabolomic profiling of the 15 mice was conducted to trace downstream variations of the altered genes. A total of 1090 metabolites were detected in the fecal samples. These metabolites can be classified into 15 classes, mainly including amino acid and its metabolites, organic acid and its derivatives, nucleotide and its metabolites, glycerophospholipids, and fatty acid.

**Fig. 3:**
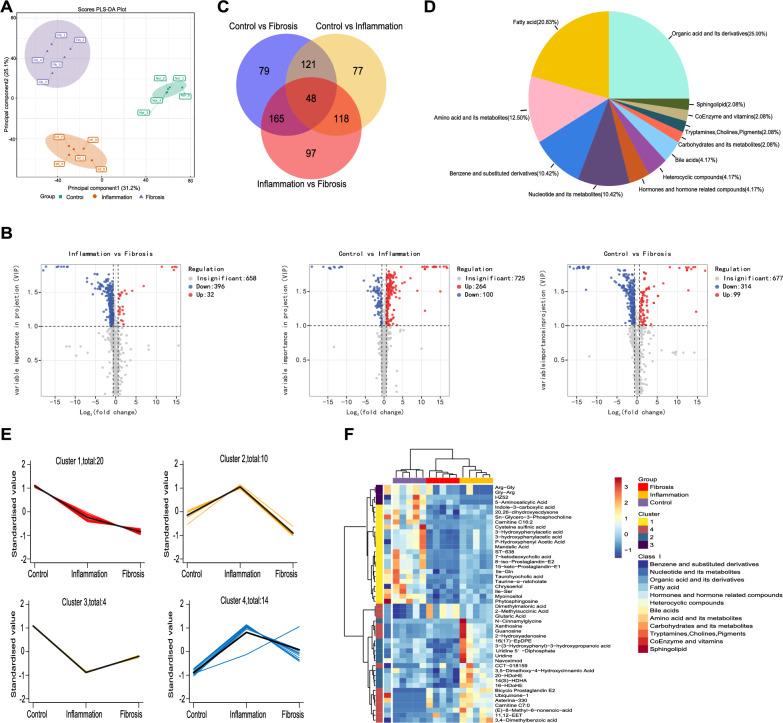
Altered metabolic profiles in the feces of the fibrosis mouse model. **A** PLS-DA multivariate statistical model of the Control, Inflammation, and Fibrosis groups. **B** Volcano plot of metabolites, with blue representing down-regulation, gray representing non-significance, and red representing up-regulation. **C** Venn diagram shows the shared or unique differential metabolites among the three groups. **D** Classification of the 48 SAMs among the three groups. Each color displays a class of metabolites, with the specific percentage highlighted in the pie chart. **E** K-means clustering diagram of the 48 SAMs among the three groups, with the specific number of metabolites presented in each cluster, and all the clusters follow the timeline of Control (Ctrl), Inflammation (TNBS-4W), and Fibrosis (TNBS-6W). **F** Cluster heatmap of the relative abundance of the 48 SAMs. *n* = 5 per group. *PLS-DA* partial least squares discriminant analysis, *SAMs* significantly altered metabolites

The PLS-DA results showed distinct trends in fecal metabolic profiles between the three experimental groups (Fig. 3A). Compared with the Control group, a total of 364 and 413 metabolites were differentially altered in the Inflammation and Fibrosis group, respectively, while 428 differential metabolites were identified between the Inflammation and Fibrosis group (|log2FC|> 0.58 and VIP ≥ 1; Fig. 3B). We identified 48 differential metabolites between all groups (Fig. 3C, Additional file 1: Table S9), among which, lipids (*e.g.*, cholines, bile acids, sphingolipid and fatty acid) accounted for 29.16%, whereas organic acids and their derivatives accounted for 25.00% (Fig. 3D). Four trends in the variation of these metabolites were retrieved via k-means cluster analysis (Fig. 3E). The metabolites in Cluster1 mainly included organic acids and their derivatives, as well as lipids, which decreased continuously in the fibrosis model (Fig. 3E, F, Additional file 1: Table S9). The metabolites in Cluster2 and Cluster4 mainly included fatty acid, organic acids and their derivatives, as well as nucleotides and their derivatives, which increased markedly in the inflammation phase but notably decreased in the fibrosis phase (Fig. 3E, F, Additional file 1: Table S9). These results implied that lipid and organic acid metabolism was fundamentally altered during fibrogenesis in mice.

### Correlations between host genes and fecal metabolites in the development of intestinal fibrosis

**Fig. 4:**
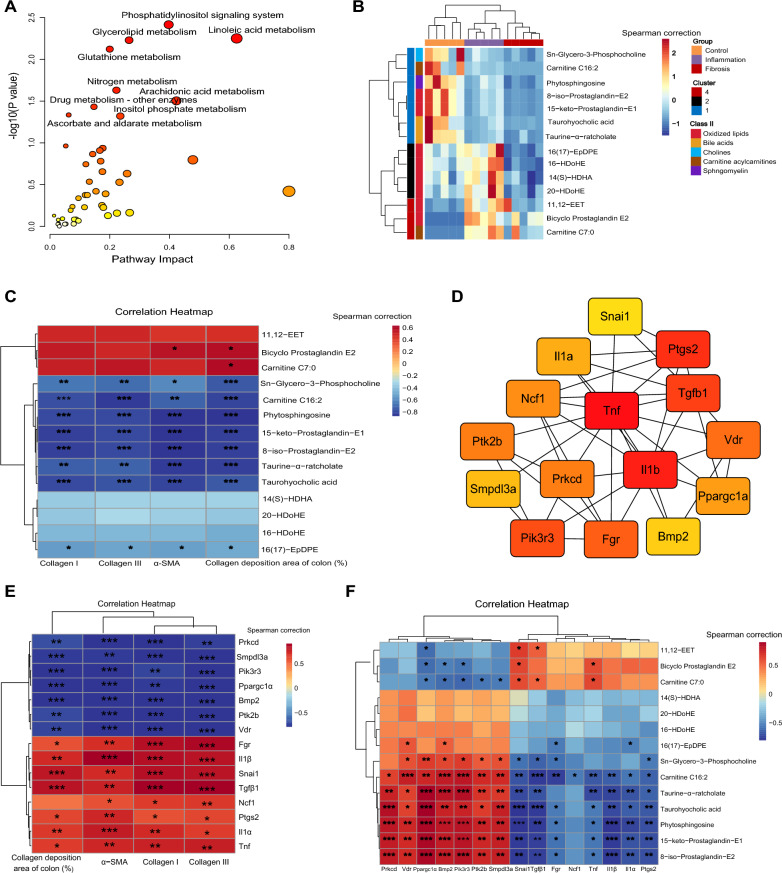
Correlations between lipid metabolism-related genes and lipid metabolites. **A** KEGG joint-pathway analysis of the DEGs in Pattern1 and the 48 SAMs; the nine metabolic pathways with *P* < 0.05 are highlighted with their names. **B** Cluster heatmap of the relative abundances of the 14 lipid metabolites screened from the 48 SAMs. **C** Spearman’s rank correlation between the 14 metabolites and 4 fibrotic indicators reflecting the degree of intestinal fibrosis in mice. **D** The top15 hub genes screened by PPI network and CytoHubba plug-in with degree values. **E** Spearman’s rank correlation between the top15 hub genes and 4 fibrotic indicators. **F** Spearman’s rank correlation between the top15 hub genes and 14 lipid metabolites. Fibrotic indicators: α-SMA, Collagen I, and Collagen III correspond to the mRNA level of α-SMA, Collagen I, and Collagen III genes, respectively, in colon samples detected by qPCR. Collagen deposition area of colon (%): quantitative results of collagen deposition determined by ImageJ software. *n* = 5 per group. *: *P* < 0.05, **: *P* < 0.01, ***: *P* < 0.001

KEGG joint pathway analysis of DEGs in Pattern1 and the above 48 SAMs was performed using the MetaboAnalyst5.0 platform. Interestingly, these genes and metabolites were mainly enriched in lipid metabolism-related pathways, such as glycerolipids, linoleic acid, and arachidonic acid metabolism (Fig. 4A), indicating that lipid metabolic disorders might be an important driving factor in the progression of intestinal fibrosis. The metabolomics analysis showed that 14 lipid metabolites were dramatically changed during the progression of intestinal fibrosis (Fig. 4B). Moreover, most of them exhibited negative correlations with fibrotic indicators (Fig. 4C), in particular, Taurine-α-ratcholate, 15-keto-Prostaglandin-E1, Carnitine C16:2, 8-iso-Prostaglandin-E2, Phytosphingosine, and Taurohyocholic acid. Among the 52 lipid metabolism-related DEGs, the top15 hub genes were identified based on PPI network analysis (Fig. 4D), namely, *Tgb1*, neutrophil cytosolic factor 1 (*Ncf1*), sphingomyelin phosphodiesterase, acid-like 3A (*Smpdl3a*), protein kinase C-delta (*Prkcd*), *Vdr*, tumor necrosis factor (*Tnf*), bone morphogenetic protein 2 (*Bmp2*), interleukin 1 beta (*Il1b*), interleukin 1 alpha (*Il1a*), phosphoinositide-3-kinase regulatory subunit 3 (*Pik3r3*), FGR proto-oncogene, Src family tyrosine kinase (*Fgr*), prostaglandin-endoperoxide synthase 2 (*Ptgs2*), *Ppargc1a*, *Snai1*, and PTK2 protein tyrosine kinase 2 beta (*Ptk2b*). Among them, seven (*Prkcd*, *Smpdl3a*, *Pik3r3*, *Ppargc1a*, *Bmp2*, *Ptk2b*, and *Vdr*) and eight (*Fgr*, *Il1b*, *Snai1*, *Tgfb1*, *Ncf1*, *Ptgs2*, *Il1a*, and *Tnf*) genes were negatively and positively correlated with fibrotic indicators, respectively (Fig. 4E). Our findings suggested that the development of intestinal fibrosis was closely related to unique metabolic signatures, including the lipid metabolic disorders.

Using Spearman’s correlation analysis, we also identified the most significant host gene–metabolite associations (Fig. 4F, Additional file 1: Table S10). Among the strongest correlations (|R|> 0.8 and *P* < 0.05), *Ppargc1a* presented the highest positive correlations with five lipids (Phytosphingosine, 8-iso-Prostaglandin-E2, 15-keto-Prostaglandin-E1, Taurine-α-ratcholate, and Taurohyocholic acid); *Pik3r3* presented positive correlations with five lipids (8-iso-Prostaglandin-E2, Taurine-α-ratcholate, 15-keto-Prostaglandin-E1, Phytosphingosine, and Carnitine C16:2); *Snai1* and *Il1b* showed negative correlations with Phytosphingosine while *Prkcd* and *Bmp2* showed positive correlations with Phytosphingosine; *Bmp2* showed a positive correlation with Taurine-α-ratcholate; and *Snai1* showed a negative correlation with Taurohyocholic acid. Collectively, changes in the host genes of TNBS-treated mice were closely related to the disturbance in fecal metabolites. Noteworthy, the decrease in *Ppargc1a* in the intestinal fibrosis model might be an especially important factor in causing metabolic alterations and disease progression.

### Potential diagnostic function of fecal metabolites

**Fig. 5:**
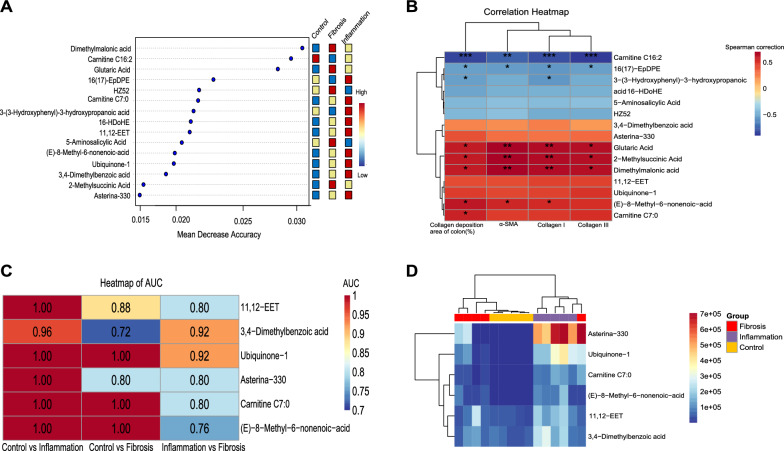
Screening for potential metabolic biomarkers of intestinal fibrosis in mice. **A** Top15 metabolites screened by the random forest algorithm after standardized correction according to the prediction accuracy of sample grouping, with red representing high expression and blue representing low expression. **B** Spearman’s rank correlation between the top15 metabolites and 4 fibrotic indicators, which reflect the degree of intestinal fibrosis in mice. **C** AUC heatmap of the top15 metabolites (only six metabolites with AUC > 0.7 among the three groups). **D** Heatmap of the relative abundance of the six metabolites screened by the random forest algorithm and ROC curves. Fibrotic indicators: α-SMA, Collagen I, and Collagen III correspond to the mRNA level of α-SMA, Collagen I, and Collagen III genes, respectively, in colon samples detected by qPCR. Collagen deposition area of colon (%): quantitative results of collagen deposition determined by ImageJ software. *n* = 5 per group. *: *P* < 0.05, **: *P* < 0.01, ***: *P* < 0.001. *KEGG* Kyoto Encyclopedia of Genes and Genomes, *AUC* area under the curve, *ROC* receiver operating characteristic

We also found that several differentially abundant metabolites might be markers for the diagnosis of intestinal fibrosis. The top15 metabolites were screened using a random forest machine learning model, and they were expected to be useful in identifying intestinal fibrosis (Fig. 5A). A Spearman’s correlation matrix was generated to assess the correlations between these differential metabolites and fibrotic indicators. Among them, certain important metabolites (*e.g.*, 16(17)-EpDPE and Carnitine C16:2) were negatively correlated with the degree of intestinal fibrosis, whereas others (*e.g.*, 2-Methylsuccinic Acid, Glutaric Acid, and Dimethylmalonic acid) were positively correlated (Fig. 5B). Prediction models of the Control group versus the Inflammation group, the Control group versus the Fibrosis group, and the Inflammation group versus the Fibrosis group were established respectively to assess how fecal metabolite composition changed in regards to the degree of intestinal fibrosis via the machine learning algorithm and ROC analysis. The ROC analysis results were presented in the Additional file 1: Table S11. Ultimately, six metabolites were associated with the status of intestinal fibrosis with an area under the ROC curve (AUC) greater than 0.7, including 11,12-EET, 3,4-Dimethylbenzoic acid, Ubiquinone-1, Asterina-330, Carnitine C7:0, and (E)-8-Methyl-6-nonenoic-acid (Fig. 5C). A cluster analysis heatmap further confirmed that intestinal fibrotic status could be distinguished from normal status based on fecal metabolites (Fig. 5D). These results implied that certain important lipids and organic acids metabolites, including 11,12-EET, Carnitine C7:0 and (E)-8-Methyl-6-nonenoic-acid, might serve as potential metabolic biomarkers of colitis-related intestinal fibrosis in the mouse model.

## Discussion

In this study, we investigated the changes of gene expression and metabolite abundance in a TNBS-induced intestinal fibrosis mouse model at different time points using colonic RNA-seq and fecal targeted metabolomics techniques. Tendency and enrichment analyses showed that 679 DEGs with enduring changes were primarily enriched in immune response-related signaling pathways and metabolism-related biological processes. Noticeably, 15 hub genes related to lipid metabolism were found to closely correlate with the development of intestinal fibrosis. We found that the formation of intestinal fibrosis was accompanied by marked fecal metabolic disturbances. Among the 48 SAMs, 14 lipid metabolites were dramatically altered during the development of intestinal fibrosis. Integrated analysis of the transcriptomics and metabolomics implied that perturbations of lipids were strongly associated with enduring alterations in lipid metabolism-related genes. In addition, six metabolites were considered as potential biomarkers of intestinal fibrosis in mice. Collectively, our results indicate that intestinal fibrosis in colitis mice might be related to dysregulated lipid metabolism and host-metabolism interactions.

Various signaling pathways are involved in fibrotic formation through myriad complex interactions and signaling cascades [35]. In this study, we observed that the DEGs were functionally annotated in immune response-related KEGG pathways, including the PI3K-Akt, MAPK, NF-kappa B, and JAK-STAT signaling pathways. A number of studies have shown that persistent or dysregulated signaling pathways, such as the MAPK, PI3K-Akt, and JAK-STAT pathways, contribute to renal and pulmonary fibrosis via regulating cell proliferation, differentiation, apoptosis, and extracellular matrix accumulation [35–37]. Additionally, specific blockade of the NF-kappa B and PI3K-Akt signaling pathways could potently protect against colitis-associated intestinal fibrosis [38, 39]. These studies confirm that dysregulated immune response-related signaling pathways might play an important role in the onset of intestinal fibrosis. Mechanistic studies have shown that pharmacologic targeting of immune-related signaling pathways could effectively alleviates colitis in animal models and individuals with IBD [40, 41]. Therefore, we can develop drugs that target interventions for these important specific pathways to prevent and treat intestinal fibrosis.

Moreover, the 15 hub genes that were identified in the PPI network might play a key role in causing intestinal fibrosis. Among the eight continuously up-regulated genes, the well-known pro-fibrotic factors *Tgfb1* and *Il1b*, exhibited the strongest positive correlations with the degree of intestinal fibrosis. Studies had proved that the transcriptional levels of *Il1b* and *Tgfb1* are up-regulated in the mucosa, submucosa, and muscle of narrow intestines in IBD patients [34]. However, the precise mechanisms of *Tgfb1* and *Il1b* in intestinal fibrosis warrant further investigation. Among the seven genes that exhibited a continuous downward trend, *Vdr*, *Bmp2*, and *Ppargc1a* showed the most marked negative correlation with the degree of intestinal fibrosis. *Vdr*, a member of the nuclear receptor superfamily, is a key molecule in genetic regulation, immunomodulation, inflammation control, and microbiota regulation [42]. Both in vitro and in vivo, *Vdr* activation can alleviate intestinal fibrosis by inhibiting abnormal fibroblast activation and migration, as well as epithelial mitochondria-mediated EMT [43–45]. *Bmp2*, a member of the TGF-β superfamily, is involved in fibrosis development of a variety of tissues and cells through the Smad signaling pathway [46, 47]. Additionally, *Bmp2* functions as a negative regulator of organ fibrogenesis by antagonizing TGF-β1-induced profibrogenic signals [48, 49]. Hence, *Bmp2* might also play an important role in intestinal fibrosis. *Ppargc1a* induction is beneficial in maintaining mitochondrial integrity, enhancing intestinal barrier function, and decreasing colitis [50, 51], which might help to prevent chronic colitis-associated intestinal fibrosis. Additionally, mechanistic studies have revealed that the restoration of *Ppargc1a* activity protects against kidney fibrosis by restoring mitochondrial viability and dynamics and reversing fatty acid oxidation defects [52, 53]. Accordingly, we speculated that continually down-regulated *Ppargc1a* might be an important factor in the formation of intestinal fibrosis.

Lipid plays vital importance in affecting cell membranes, metabolic processes and signaling pathways, and acting as energy storage sources. Lipid metabolism disorders have been reported in the serum, plasma, urine, feces, and colonic mucosa samples of IBD patients [54]. Nevertheless, few studies have focused on the metabolite profiles of IBD patients with intestinal fibrosis. In this study, we found that metabolite disturbances in mouse feces, especially lipid and organic acid metabolites, were closely associated with intestinal fibrosis progression. We found that Phytosphingosine continually decreased during the progression of intestinal fibrosis, which was previously reported to perform anti-inflammatory activity in cell-based assays and ameliorate acute colitis in mice [55, 56]. We also found arachidonic acid metabolism products such as 11,12-EET and prostaglandin E2, was decreased in the fibrosis phase, which have exhibited certain anti-fibrotic activities in other organ [57–60]. Therefore, we speculated that supplementation with substances with anti-inflammatory and anti-fibrotic activities during the inflammation phase may help to mitigate or prevent intestinal fibrosis. However, the relationship between the lipid metabolites identified in our study and fibrotic diseases have rarely been reported, possibly because previous studies have only focused on differential metabolites after fibrotic formation. Our study explored changes in metabolites during the progression of inflammation-associated intestinal fibrosis, and these significantly altered metabolites might favor the early recognition of fibrosis.

The close connections between lipid metabolism-related genes and metabolites might contribute to the development of intestinal fibrosis. On the one hand, bioactive metabolites especially the lipid metabolites could drive key modification processes for DNA, RNA and proteins to regulate fundamental biological processes of IBD development, such as signal transduction, protein balance, and gene expression regulation [10, 12]. On the other hand, recent studies have revealed that alterations in lipid metabolic processes, especially the fatty acid metabolism, are common mechanisms and central pathophysiological pathways for the development of various fibrotic diseases [11]. We have identified four DEGs strongly correlated with lipid metabolites, including *Bmp2*, *Ppargc1a*, *Pik3r3*, and *Snai1*. *Ppargc1a* plays a pivotal role in lipid and metabolic regulation in many vital organs, including adipose tissue, skeletal muscle, heart, liver, and kidney [61]. *Bmp2* is likely to induce adipogenesis by promoting the expression of lipoxygenase (*LOX*) and PPAR gamma (*PPARγ*) in preadipocytes [62]. Yang et al. [63] reported that *Pik3r3* regulates PPAR alpha (*PPARα*) expression to stimulate fatty acid β-oxidation. Studies have suggested that adipose *Snail1* acts as an epigenetic rheostat that governs lipid metabolism and partitioning between tissues [64, 65]. Furthermore, genetic alterations or pharmacologic targeting of altered lipid metabolic processes have great potential to inhibit fibrosis development [11]. Thus, more mechanistic studies are required to investigate the role of the interaction between these DEGs and lipid metabolism in the pathogenesis of intestinal fibrosis.

We have conducted time series analyses to better identify the dynamic characteristics of gene regulatory and metabolites fluctuate network models, which largely compensates for the fact that clinical samples cannot dynamically track intestinal fibrosis. To the best of our knowledge, this is the first comprehensive dynamic transcriptomics and metabolomics analysis of colitis-associated intestinal fibrosis. However, we acknowledge certain limitations in our study. First, we currently conduct fecal metabolomics studies with limited samples, but many factors can influence the abundance of metabolites. So, adding more biological replicates for each group and detecting serum metabolites might greatly help to increase the reliability and persuasiveness of our findings and better explore metabolic changes associated with fibrosis. Second, we can’t cross-validate our findings with external data, because similar longitudinal studies for chronic inflammation-associated intestinal fibrosis are currently lacking. Third, we did not characterize the precise underlying mechanism for the development of intestinal fibrosis, which must be addressed in more detailed and comprehensive studies. Finally, we need to perform colonic transcriptomic and fecal metabolomic of IBD patients before and after intestinal fibrosis to further confirm our findings. However, it is inconvenient to dynamically track the progression of intestinal fibrosis in patients because the fibrogenesis processes have already been established when fibrosis is detected. Thus, in a future study, we will try to collect intestinal biopsies and fecal samples from patients with intestinal fibrosis to verify our results.

## Conclusions

We comprehensively evaluated dynamic genetic and metabolic alterations during the development of intestinal fibrosis. Intestinal fibrosis in colitis mice might be related to dynamic changes in gene expression and metabolites. Moreover, lipid metabolism disorders might be significant factors in the development of intestinal fibrosis. These findings could provide new insights into the pathogenesis of intestinal fibrosis.

### Supplementary Information

**Additional file 1: Table S1**: Primer sequences for quantitative PCR (qPCR). **Table S2**: The 2370 differentially expressed genes among three groups. **Table S3**: The results of STEM analysis. **Table S4**: The 1839 differentially expressed genes in the 6 significant time-course profiles. **Table S5**: The 82 transcription factors (TFs) in the 6 significant time-course profiles. **Table S6**: The 109 transcription cofactors (TcoFs) in the 6 significant time-corse profiles. **Table S7**: The 52 differentially expressed genes enriched in the lipids metabolism-related biological processes. **Table S8**: The top20 signaling-related and metabolism-related biological processes in Pattern1. **Table S9**: The 48 differentially altered metabolites among three groups. **Table S10**: The Spearman correlation analysis result of the 15 hub genes and 14 lipids metabolites. **Table S11**: The ROC analysis result of the 48 differentially altered metabolites.

## Data Availability

The datasets used and analysed during the current study are available from the corresponding author on reasonable request.

## Abbreviations

IBD: Inflammatory bowel disease
CD: Crohn’s disease
UC: Ulcerative colitis
TNBS: 2,4,6-Trinitrobenzene sulfonic acid
NOD2: Nucleotide-binding oligomerization domain 2
TLR4: Toll-like receptors-4
TL1A: Tumor necrosis factor-like ligand 1A
RNA-seq: RNA-sequencing
SUCNR1: Succinate receptor
qPCR: Quantitative polymerase chain reaction
H&E: Hematoxylin and eosin
FPKM: Fragments per kilobase of exon per million mapped reads
STEM: Short Time-series Expression Miner
DEGs: Differentially expressed genes
TFs: Transcription factors
TcoFs: Transcription cofactors
GO: Gene Ontology
KEGG: Kyoto Encyclopedia of Genes and Genomes
VIP: Variable importance projection
PLS-DA: Partial least squares discriminant analysis
FC: Fold-change
SAMs: Significantly altered metabolites
ROC: Receiver operating characteristic
PPI: Protein–protein interaction
SEM: Standard error of mean
PI3K-Akt: Phosphatidylinositol 3-kinase/protein kinase B
MAPK: Mitogen-activated protein kinase
JAK-STAT: Janus kinase/signal transducers and activators of transcription
Foxo3: Forkhead box O3
Myc: Myelocytomatosis oncogene
Dgkq: Diacylglycerol kinase, theta
TNF: Tumor necrosis factor
IL-17: Interleukin-17
Vdr: Vitamin D receptor
Ppargc1a: Peroxisome proliferator-activated receptor gamma coactivator 1-alpha
Bmp2: Bone morphogenetic protein 2
Tgfb1: Transforming growth factor, beta 1
Ncf1: Neutrophil cytosolic factor 1
Smpdl3a: Sphingomyelin phosphodiesterase, acid-like 3A
Prkcd: Protein kinase C-delta
Tnf: Tumor necrosis factor
Il1b: Interleukin 1 beta
Il1a: Interleukin 1 alpha
Pik3r3: Phosphoinositide-3-kinase regulatory subunit 3
Fgr: FGR proto-oncogene
Src: Family tyrosine kinase
Snai1: Snail family zinc finger 1
Ptk2b: PTK2 protein tyrosine kinase 2 beta
AUC: Area under the ROC curve
LOX: Lipoxygenase
EMT: Epithelial-mesenchymal transition
LOX: Lipoxygenase
PPARα/γ: Peroxisome proliferator-activated receptor alpha/gama
